# Case Report: Imaging findings in sneaky subungual amelanotic melanoma

**DOI:** 10.3389/fmed.2025.1686909

**Published:** 2025-11-18

**Authors:** Mei Shi, Mengyao Zhao, Xiaolu Zhu, Xixi Sun, Xiaomin Dai, Zeyang Dong, Bin Huang

**Affiliations:** 1 Department of Ultrasound, Zhejiang Hospital, Hangzhou, China; 2 The Second School of Clinical Medicine, Zhejiang Chinese Medical University, Hangzhou, China; 3 Department of Pathology, Zhejiang Hospital, Hangzhou, China

**Keywords:** subungual ultrasound, amelanotic melanoma, subungual telangiectatic granuloma, skin tumor, case report

## Abstract

We report a rare case of solitary subungual malignant melanoma in a female patient who presented with a misaligned nail without accompanying pigmentary changes. The absence of typical clinical and imaging features delayed definitive diagnosis. Both magnetic resonance imaging (MRI) and ultrasound (US) initially suggested a benign lesion, favoring subungual telangiectatic granuloma. However, histopathology ultimately confirmed subungual melanoma with negative Human Melanoma Black-45 (HMB-45) immunostaining. Amelanotic melanomas are particularly prone to misdiagnosis or delayed recognition due to their lack of visible pigmentation. We present a detailed analysis of the imaging and pathological findings, highlighting specific ultrasound characteristics, with the aim of providing a valuable reference for the clinical diagnosis and management of such uncommon cases.

## Introduction

Subungual melanoma (SUM), a rare variant of acral melanoma, predominantly occurs in the thumb and great toes of adults aged 50–70 years (1). Amelanotic melanoma, an uncommon histologic subtype, constitutes a small proportion of all melanomas. While it most commonly occurs in sun-exposed regions such as the ears, nose, and face, it is far less frequent in the subungual area (2). Currently, the primary diagnostic modalities for subungual melanoma include dermoscopy, magnetic resonance imaging, ultrasound examination, and pathological biopsy. We present a case of subungual amelanotic melanoma, where its atypical clinical presentation and Human Melanoma Black-45 (HMB-45) negativity pose diagnostic challenges, ultimately leading to a misdiagnosis of subungual telangiectatic granuloma. This case report was conducted in accordance with ethical standards. Written informed consent was obtained from the patient and approved by the Ethics Committee of Zhejiang Hospital (Ethics Approval No. 2025-CA-34).

## Case description

**Figure 1:**
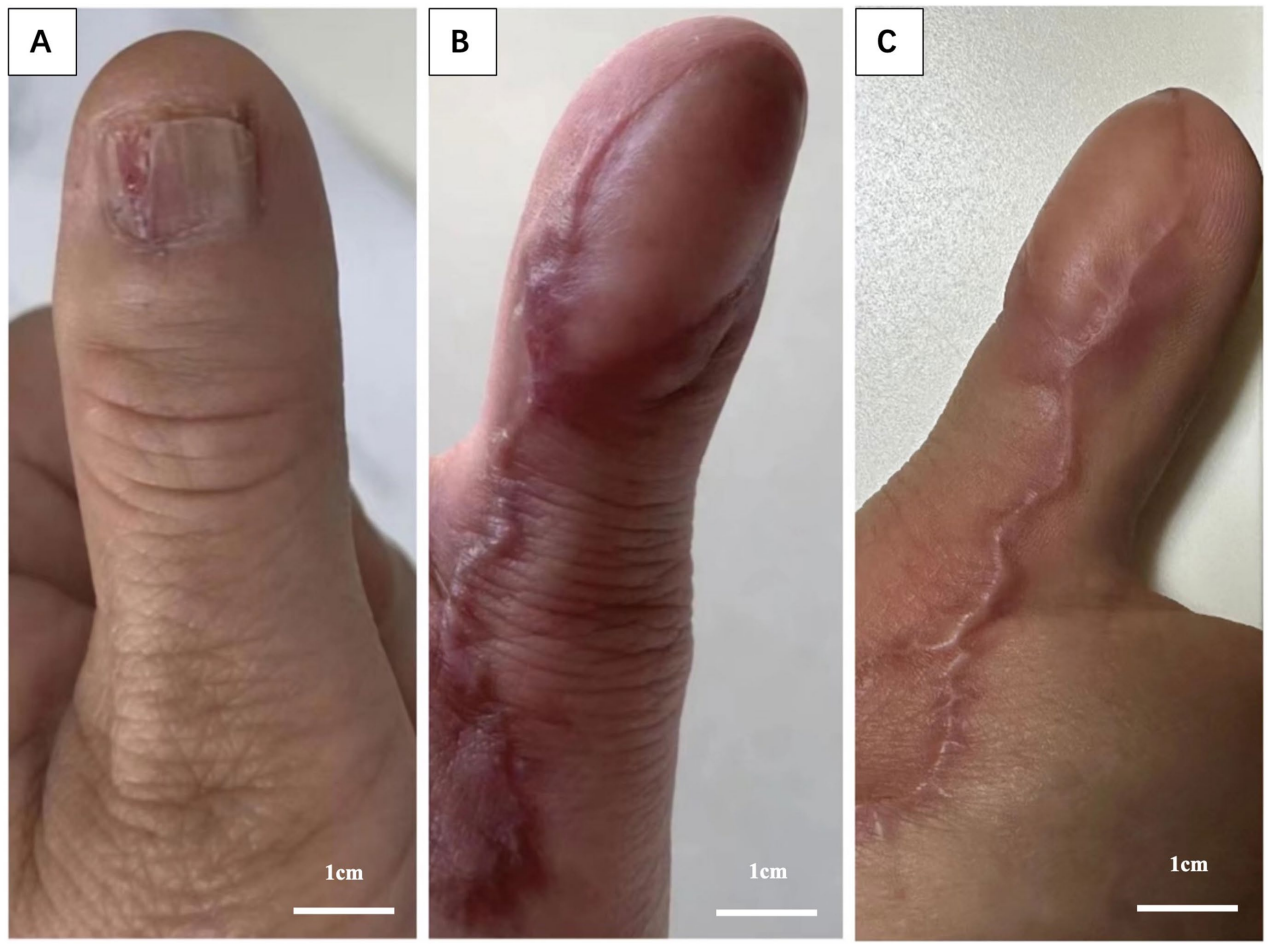
Gross features. **(A)** Longitudinal splitting and bleeding of the left thumb nail, with a rice-like mass observed between the nail splits. **(B)** No signs of tumor recurrence in the left thumb, 6 months post-surgery. **(C)** No signs of tumor recurrence in the left thumb, 2 years post-surgery.

A 52-year-old woman presented to the dermatology department with complaints of longitudinal splitting and bleeding of her left thumbnail, without any history of trauma. The distal portion of the left thumbnail appeared swollen, and a rice-sized mass was observed between the split nails, which was mildly tender to the touch. Neither the epidermis nor the nail showed any blackening or discoloration (Figure 1). She had been treated with topical antifungal lacquer for 2 months at a local hospital for a misaligned nail that had persisted for 1 year.

**Figure 2:**
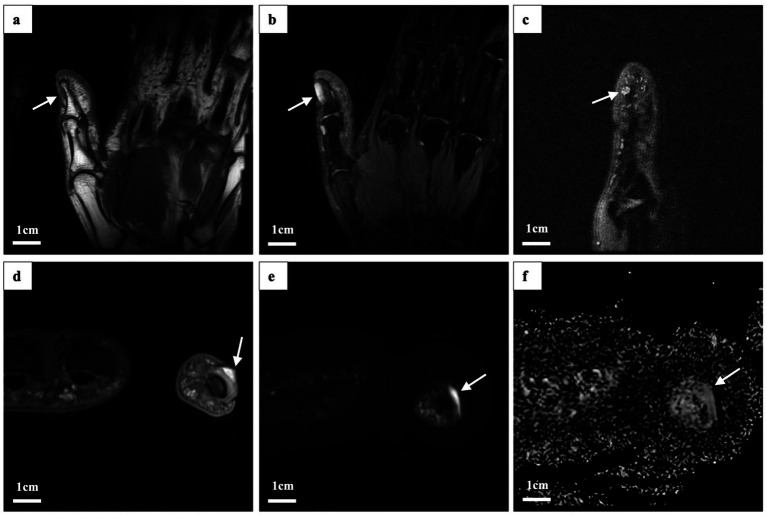
Magnetic resonance imaging findings. The lesion (indicated by the white arrow) exhibited the following characteristics: **(a)** Low to moderate signal intensity on oblique coronal (OCor) T1-weighted imaging (WI) fast spin echo. **(b)** High signal intensity on oblique coronal T2-weighted imaging (T2WI) and fat-suppressed proton density (fsPD). **(c)** High signal intensity in oblique sagittal T2WI fsPD. **(d)** High signal intensity in oblique axial (OAx) T2WI fsPD. **(e)** High signal intensity in OAx diffusion-weighted imaging (DWI). **(f)** Low signal intensity on the apparent diffusion coefficient (ADC) map.

**Figure 3:**
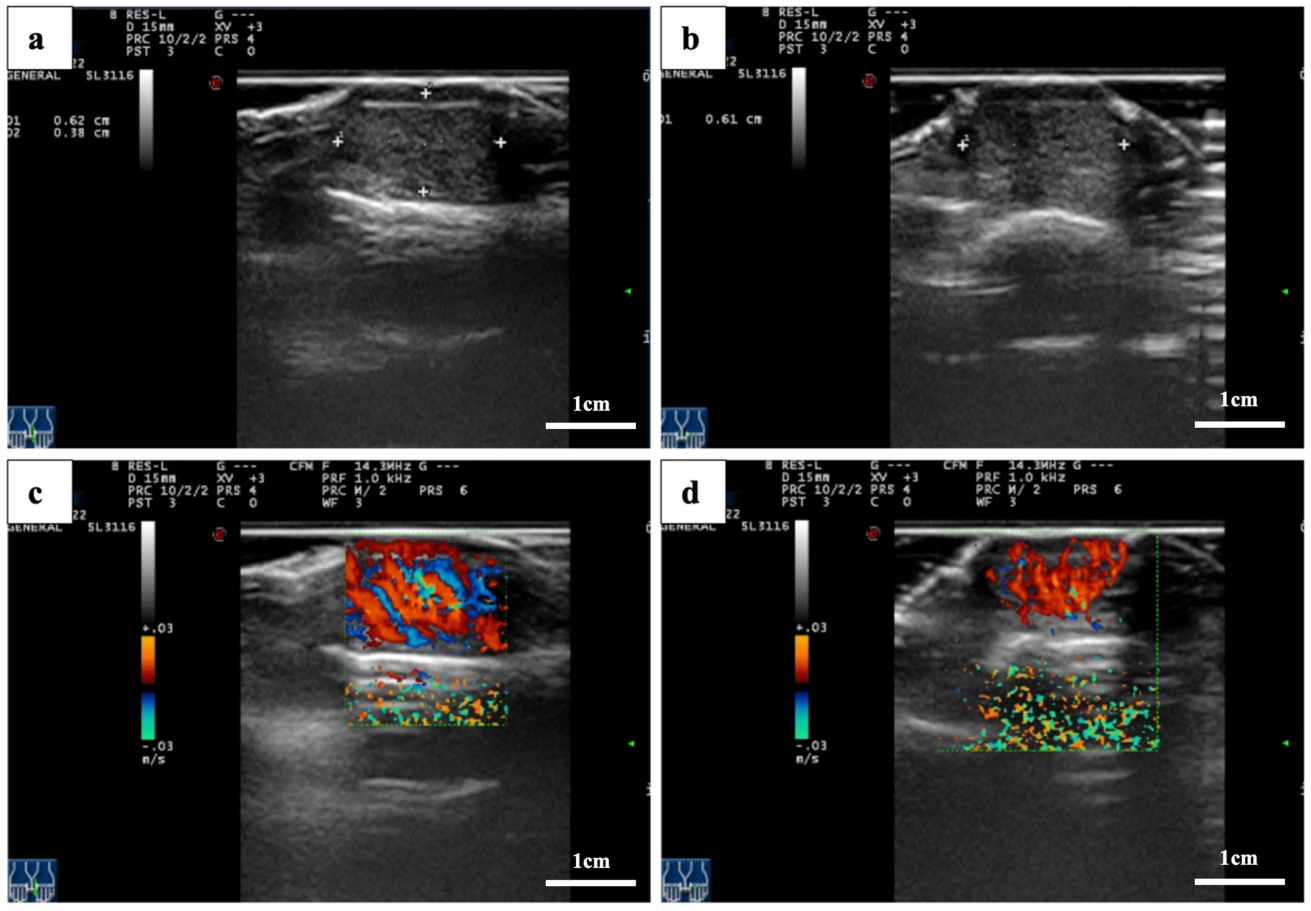
Ultrasound images. **(a,b)** A hypoechoic nodule with well-defined borders is located beneath the thumbnail. **(c,d)** Color Doppler flow images showing nodular microvascular enrichment.

Magnetic resonance imaging (MRI) of the left hand revealed a flaky abnormal signal shadow on the dorsal side of the first distal phalanx. The lesion was low to moderate signal on T1-weighted imaging (T1WI), high signal on T2-weighted imaging (T2WI), and T2 fat suppression sequences (FSE) with well-defined borders. High signal with poorly defined borders on DWI sequences. The surrounding soft tissue was edematous, but there was no obvious destruction of the bone (Figure 2). High-frequency ultrasonography (14.3 MHz) revealed a well-defined low-echo nodule measuring approximately 0.62 × 0.38 × 0.61 cm, exhibiting highly abundant blood flow signals in color Doppler flow imaging (CDFI) (Figure 3).

**Figure 4:**
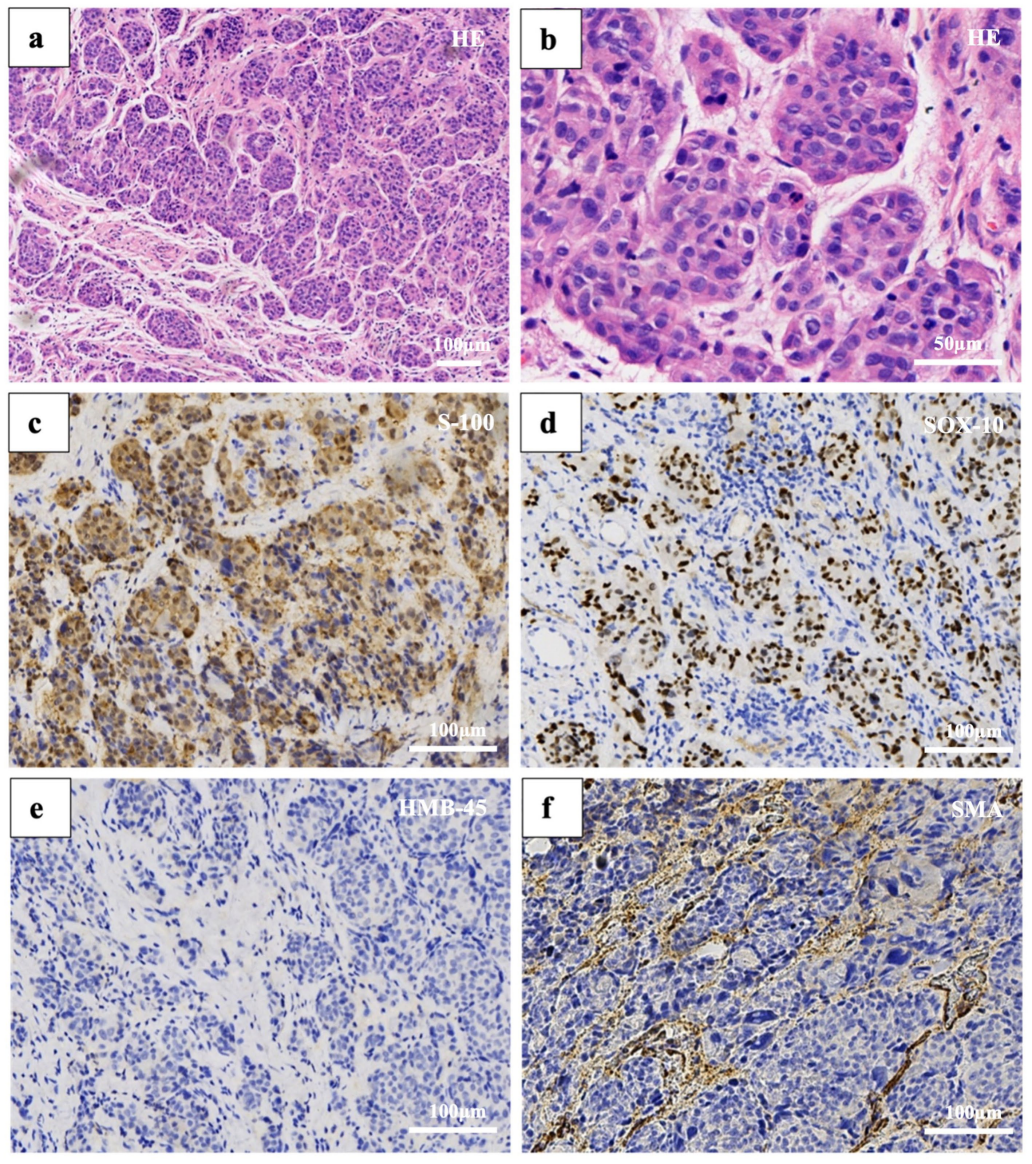
Histopathology. Hematoxylin and eosin staining reveals: **(a)** Atypical oval cells aggregated in patches or nests (50× magnification). **(b)** Large cells with high nuclear-to-cytoplasmic ratios, deeply stained, without obvious necrosis or pigment deposition (200× magnification). Immunohistochemical staining: **(c)** S-100 (100× magnification). **(d)** SOX-10 (100× magnification). **(e)** HMB-45 (100× magnification). **(f)** Smooth muscle actin (100× magnification).

We first considered benign lesions, favoring subungual telangiectatic granuloma. Subsequently, the patient underwent a local tissue biopsy. However, histopathological analysis confirmed a diagnosis of melanoma. The lesion showed diffusely infiltrative tumor cells in the fibrous tissue in patches or nests, which were relatively uniform in size, with a large, deeply stained nucleus, obvious atypia, and rare mitosis. No clear necrosis or pigment deposition was observed. Immunohistochemistry was positive for S-100 and SOX10 and negative for HMB45, smooth muscle actin, creatine kinase, and CD34 (Figure 4). Furthermore, the negative results for EMA, CD31, desmin, GATA3, PAX8, ER, PR, and P63 effectively rule out epithelial, vascular, smooth muscle-derived, and breast cancer tumors to a certain extent. Following clinical and pathological evaluation, the patient underwent extended resection of the local tumor without amputation, radiation, or chemotherapy, due to Breslow thickness of 2.1 mm and Clark level IV. The thumb was reconstructed using an autologous flap, which successfully preserved the distal phalanx. No recurrence was observed at 2-year postoperative follow-up, and the patient did not require adjuvant therapy.

At the 2-year follow-up, the patient demonstrated excellent functional and cosmetic outcomes. Tip pinch strength was 85% of the contralateral hand, with the affected left thumb exhibiting 3.6 kg of strength compared to 4.5 kg on the right. Thumb opposition was nearly complete, with a Kapandji score of 9/10, allowing the patient to perform fine motor activities, such as buttoning and flipping pages, without difficulty. The surgical scar was minimally noticeable, with a Vancouver Scar Scale score of 3, and the patient reported a high level of satisfaction with the aesthetic result (9/10 on a numerical rating scale) (Figure 1).

## Discussion

Subungual melanoma (SUM), a rare variant of acral melanoma, accounts for 1.0–1.7% of cutaneous melanomas in Europe and 7.1–29.3% of all melanomas in Asia (3, 4). Due to its high malignancy, subtle clinical presentation, and difficulty of early diagnosis, distant metastasis is common, which often results in poor prognosis (5). Early detection and complete surgical excision remain the cornerstone for achieving a curative outcome in all types of melanoma. Delayed diagnosis is associated with increased Breslow depth, a higher risk of metastasis, and reduced survival. Amelanotic melanoma accounts for only a small proportion of all melanomas, and its occurrence in the subungual region is and is particularly uncommon in the subungual region (5). Its rarity, combined with the absence of pigmentation, significantly increases the risk of misdiagnosis. Additionally, studies have shown that, compared to pigmented melanoma, non-pigmented melanoma tends to have fewer specific white blood cells that identify and attack the tumor, which may suggest a poorer prognosis (6).

The diagnosis of subungual melanoma presents numerous challenges. While extensive literature documents such cases, the present case exhibits several distinctive features that set it apart from previously reported instances (7–10). First, acral amelanotic melanoma is rare, especially when it initially manifests in changes in nail morphology. Second, this case exhibits negative HMB-45 expression, which is also relatively uncommon as a highly specific indicator. Finally, detailed imaging data are presented, an aspect seldom emphasized in earlier reports.

Our case presented an unusual immunohistochemical profile—negative HMB-45 expression—which is particularly uncommon and may further obscure the correct diagnosis. HMB-45 is a highly specific immunohistochemical marker for melanoma. Multiple studies report that 56.3 to 77% of melanomas are HMB-45 positive, with a notably high prevalence also observed in amelanotic melanoma (11, 12). Although HMB-45 is widely used as a melanocytic marker, it may be absent in certain histologic variants or tumors with minimal melanin synthesis (13, 14). Relevant literature suggests that expression levels and gradients of HMB-45 are associated with the degree of differentiation and invasiveness in melanoma (15, 16). Therefore, when HMB-45 is negative, a broader immunohistochemical panel, including S-100, SOX10, creatine kinase, smooth muscle actin, and Melan-A, should be considered to ensure diagnostic accuracy (17, 18).

Clinically, subungual amelanotic melanoma often lacks specific or pathognomonic features during its early stages. This can cause patients to underestimate the significance of their symptoms and delay medical consultation. In our patient, the earliest presentation was misalignment of nail growth, likely caused by tumor invasion of the nail matrix. This clinical presentation initially raised dermatological concern without immediate suspicion of malignancy, but early histopathological evaluation ultimately established the definitive diagnosis. Fortunately, early pathological evaluation led to an accurate diagnosis, with a Breslow thickness of 2.1 mm and Clark level IV, and a negative sentinel lymph node biopsy, allowing for a procedure that preserved both hand function and cosmetic appearance.

Radiologic evaluation of small or atypical tumors of the hand remains challenging. While MRI is an important modality for assessing the extent of soft tissue lesions, its diagnostic sensitivity to melanoma decreases when melanin content is low. Melanin shortens both T1 and T2 relaxation times, which produces a characteristic high signal on T1-weighted and low signal on T2-weighted sequences. In amelanotic melanoma, these features are absent, resulting in non-specific MRI findings (19–21). In our case, MRI did not detect the melanoma, most likely due to the absence of melanin pigmentation and the small size of the tumor.

In contrast, high-frequency ultrasound may provide superior resolution in this case. Ultrasound provides high spatial resolution for superficial lesions and allows real-time evaluation of tumor vascularity using CDFI. Upon reviewing and analyzing the images, we observed that extremely hypoechoic areas of the tumor based on two-dimensional images, with CDFI demonstrating pedicle-like vascular flow signals. These findings suggest an unbalanced and disorganized tumor blood supply, which is more characteristic of malignant lesions than benign subungual masses.

Comparing the two imaging modalities, high-frequency ultrasound excels in spatial resolution, making it particularly effective for evaluating superficial areas such as the subungual region. It provides detailed information on lesion size, morphology, and vascular characteristics (22, 23). However, its ability to assess deeper tissues is limited. In contrast, MRI offers a more comprehensive view of lesion depth and the surrounding structures, although its sensitivity may be reduced when detecting subtle vascular changes or small, non-pigmented lesions. Therefore, clinicians should recognize the complementary roles of these two imaging techniques in the diagnosis of subungual melanoma.

The main imaging differential in this case was subungual telangiectatic granuloma, a benign reactive lesion that arises secondary to chronic irritation, trauma, or infection (24, 25). This lesion typically appears hypervascular on Doppler imaging and hypoechoic on grayscale ultrasound. Due to these similarities, subungual amelanotic melanoma may be misdiagnosed as telangiectatic granuloma, particularly when pigmentation is absent. However, the presence of heterogeneous hypoechoic zones, irregular tumor margins, and pedicle-like vascular patterns can help differentiate melanoma from benign vascular lesions.

From a clinical management perspective, it is crucial to avoid empirical treatments that may obscure the underlying diagnosis. For example, topical antifungal agents can cause hypopigmentation of the nail or periungual tissue, potentially masking early melanoma and causing diagnostic delays. Melanoma should be suspected in nail bed masses, especially those in the thumbs or toes with microvascular enrichment and with or without hyperpigmentation, or a positive Hutchinson’s sign.

Based on our experience and a review of the literature, we recommend the following strategies to improve early detection of subungual amelanotic melanoma (26–33):

1. To maintain a high index of suspicion for persistent nail bed masses, especially those affecting the thumb or great toe, regardless of pigmentation status.
2. To utilize dermoscopy and high-frequency ultrasound as first-line imaging modalities for detailed structural and vascular assessment.
3. To perform histopathological examination early, and if HMB-45 is negative, use an extended immunohistochemical panel to improve diagnostic accuracy.
4. To consider genetic testing in diagnostically challenging cases to identify carcinogenic mutations and guide subsequent treatment.

In summary, this case underscores the diagnostic challenges of subungual amelanotic melanoma, particularly in the setting of HMB-45 negativity. It highlights the limitations of MRI for small, non-pigmented tumors and the potential advantages of high-frequency ultrasound in identifying subtle yet distinctive vascular and echotextural features. Prompt integration of imaging, pathology, and immunohistochemistry is essential to avoid misdiagnosis and to enable timely, function-preserving surgical management.

## Data Availability

The original contributions presented in the study are included in the article/supplementary material, further inquiries can be directed to the corresponding authors.
